# HypoxamiR-210 accelerates wound healing in diabetic mice by improving cellular metabolism

**DOI:** 10.1038/s42003-020-01495-y

**Published:** 2020-12-14

**Authors:** Sampath Narayanan, Sofie Eliasson Angelstig, Cheng Xu, Jacob Grünler, Allan Zhao, Wan Zhu, Ning Xu Landén, Mona Ståhle, Jingping Zhang, Mircea Ivan, Raluca Georgiana Maltesen, Ileana Ruxandra Botusan, Neda Rajamand Ekberg, Xiaowei Zheng, Sergiu-Bogdan Catrina

**Affiliations:** 1 Department of Molecular Medicine and Surgery, Karolinska Institutet, Stockholm, Sweden; 2 Department of Endocrinology and Diabetes, Karolinska University Hospital, Stockholm, Sweden; 3 Department of Nosocomial Infection Control, China Medical University, Shenyang, China; 4 Dermatology and Venereology division, Department of Medicine Solna, Center for Molecular Medicine and Ming Wai Lau Centre for Reparative Medicine, Karolinska Institutet, Stockholm, Sweden; 5 Department of Infectious Disease, China Medical University, Shenyang, China; 6 Departments of Medicine, Microbiology and Immunology, Indiana University, Indianapolis, IN USA; 7 Department of Anesthesia and Intensive Care Medicine, Aalborg University Hospital, Aalborg, Denmark; 8 Center for Diabetes, Academic Specialist Centrum, Stockholm, Sweden

**Keywords:** miRNAs, Diabetes complications

## Abstract

Wound healing is a high energy demanding process that needs a good coordination of the mitochondria with glycolysis in the characteristic highly hypoxic environment. In diabetes, hyperglycemia impairs the adaptive responses to hypoxia with profound negative effects on different cellular compartments of wound healing. miR-210 is a hypoxia-induced microRNA that regulates cellular metabolism and processes important for wound healing. Here, we show that hyperglycemia blunted the hypoxia-dependent induction of miR-210 both in vitro and in human and mouse diabetic wounds. The impaired regulation of miR-210 in diabetic wounds is pathogenic, since local miR-210 administration accelerated wound healing specifically in diabetic but not in non-diabetic mice. miR-210 reconstitution restores the metabolic balance in diabetic wounds by reducing oxygen consumption rate and ROS production and by activating glycolysis with positive consequences on cellular migration. In conclusion, miR-210 accelerates wound healing specifically in diabetes through improvement of the cellular metabolism.

## Abstract (web summary)

Narayanan et al. show that local administration of miR-210 accelerates wound healing specifically in diabetic mice. They find that miR-210 restores the metabolic balance in diabetic wounds by activating glycolysis and cellular migration. This study presents miR-210 as a potential therapeutic option to treat diabetic wound healing.

## Introduction

Wound healing is a complex process with high energy demands that involves several cellular compartments including keratinocytes, fibroblasts, endothelial and immune cells. The high energy needs are metabolically challenging since the wound environment is highly hypoxic due to reduced oxygen supply secondary to loss of vascularization. Wound healing is impaired in diabetes, and diabetic foot ulcer (DFU) represents a devastating complication of diabetes with drastic consequences for patients and society. The energy balance in diabetic wounds is even more difficult to achieve since hypoxia is more profound than in normoglycemic wounds^1^ and the cellular reactions to hypoxia are impaired in diabetes^2^ due to repressed hypoxia inducible factor-1 (HIF-1) signaling^3^. Local pharmacological reversal of the repressed HIF-1 signaling in diabetes is followed by the promotion of wound healing secondary to the improvement of several energy-demanding processes i.e. angiogenesis, dermal and epidermal regeneration^1,4^.

HIF-1 is a master regulator of oxygen homeostasis and therefore drives a large number of cellular processes. Currently, approximately 1000 human genes are identified to be directly influenced by HIF-1, which binds to the functionally cis-acting regulatory element called hypoxia response element (HRE)^5^.

MiR-210 is a unique microRNA that possesses an HRE and it is exclusively regulated in hypoxia by HIF-1 signaling^6^ and it is modulated in a dose-dependent manner by oxygen^7^. miR-210 is ubiquitously expressed and influences the expression of a cluster of genes that are involved in processes highly relevant for energy metabolism and for tissue regeneration including angiogenesis, cell proliferation/apoptosis, and metabolic adaptation^8^. It has previously been shown that miR-210 has higher expression levels at the edge of ischemic wounds and inhibits keratinocyte proliferation^9^, and that anti-miR-210 treatment could accelerate healing of ischemic wounds^10^_._ However, to our knowledge, the regulation and function of miR-210 in diabetic wound has not been investigated before.

In this study, we investigated the contribution of miR-210 for the hypoxia signature in wound healing in diabetes. We show that the induction of miR-210 is repressed in the wounds of diabetic patients and in experimental wounds of diabetic db/db mice. Local reconstitution of miR-210 improved wound healing specifically in db/db mice by restoring metabolic balance and improving processes central to wound healing.

## Results

### High glucose levels inhibit the induction of miR-210 expression in hypoxia

**Fig. 1:**
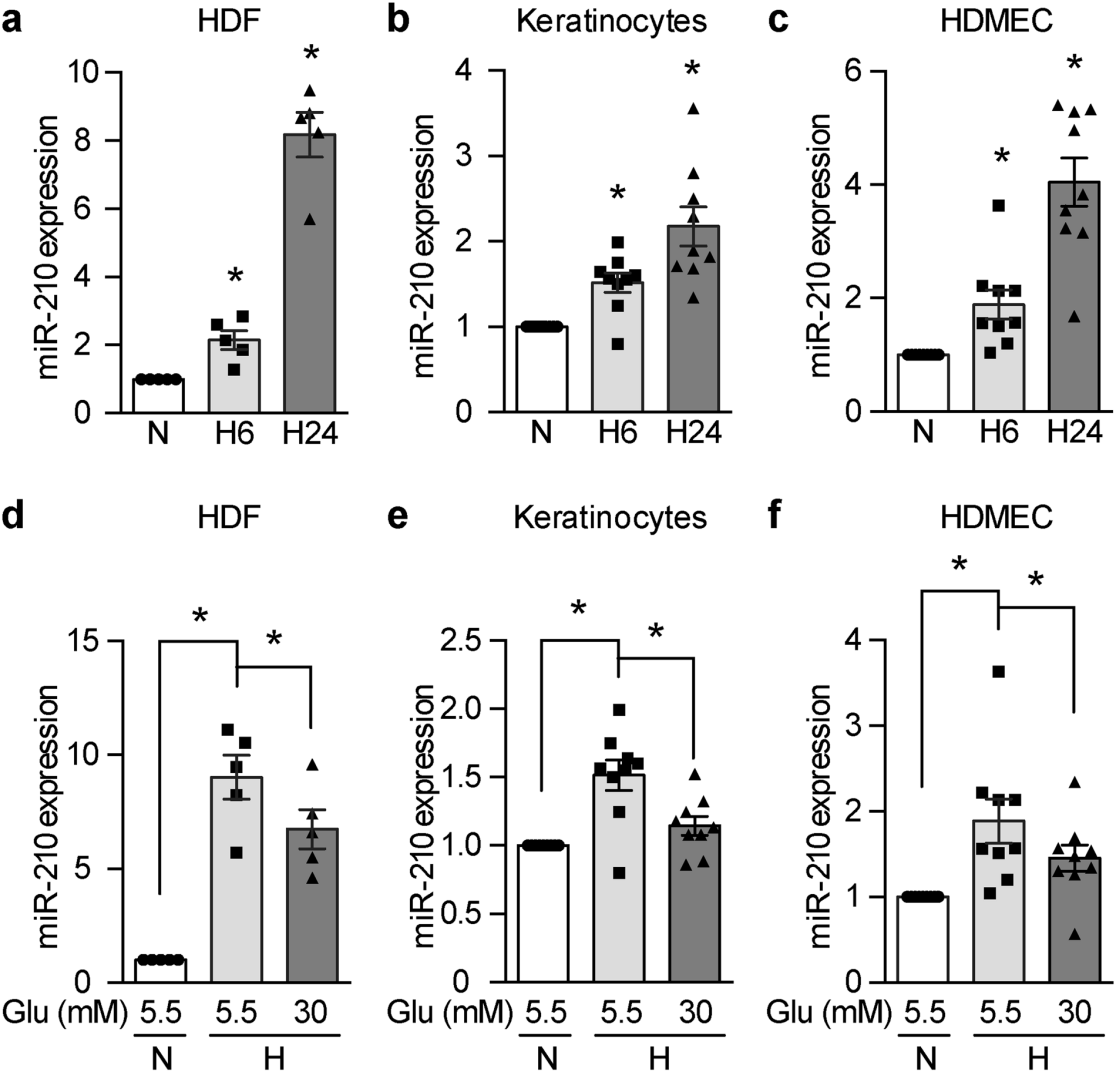
High glucose levels inhibit miR-210 induction at hypoxia. **a**–**c** miR-210 expression in human dermal fibroblasts (HDF) (*n* = 5), human dermal microvascular endothelial cells (HDMEC) (*n* = 9), and keratinocytes (*n* = 9) that were exposed to normoxia (N) or hypoxia for 6 h (H6) and 24 h (H24). *P < 0.05 compared with *N*. **d**–**f** The cells were cultured in normal (5.5 mM) or high (30 mM) glucose (Glu) levels for 24 h and exposed to normoxia (N) or hypoxia (H) for 24 h (**d**) and 6 h (**e**, **f**) respectively. Relative miR-210 levels are shown. Statistical differences were calculated by one-way ANOVA. Data are represented as mean ± s.e.m. *P < 0.05.

We first investigated the modulation of miR-210 expression by hypoxia in human dermal fibroblasts (HDF), human dermal microvascular endothelial cells (HDMEC), and keratinocytes, which are the most functionally relevant cellular components of the skin. As expected, hypoxia induced miR-210 expression in a time-dependent manner in all the three cell types (Fig. 1a–c). High glucose concentrations, however, diminished the hypoxia-induced expression of miR-210 in these cells (Fig. 1d–f).

### miR-210 expression is inhibited in diabetic wounds

**Fig. 2:**
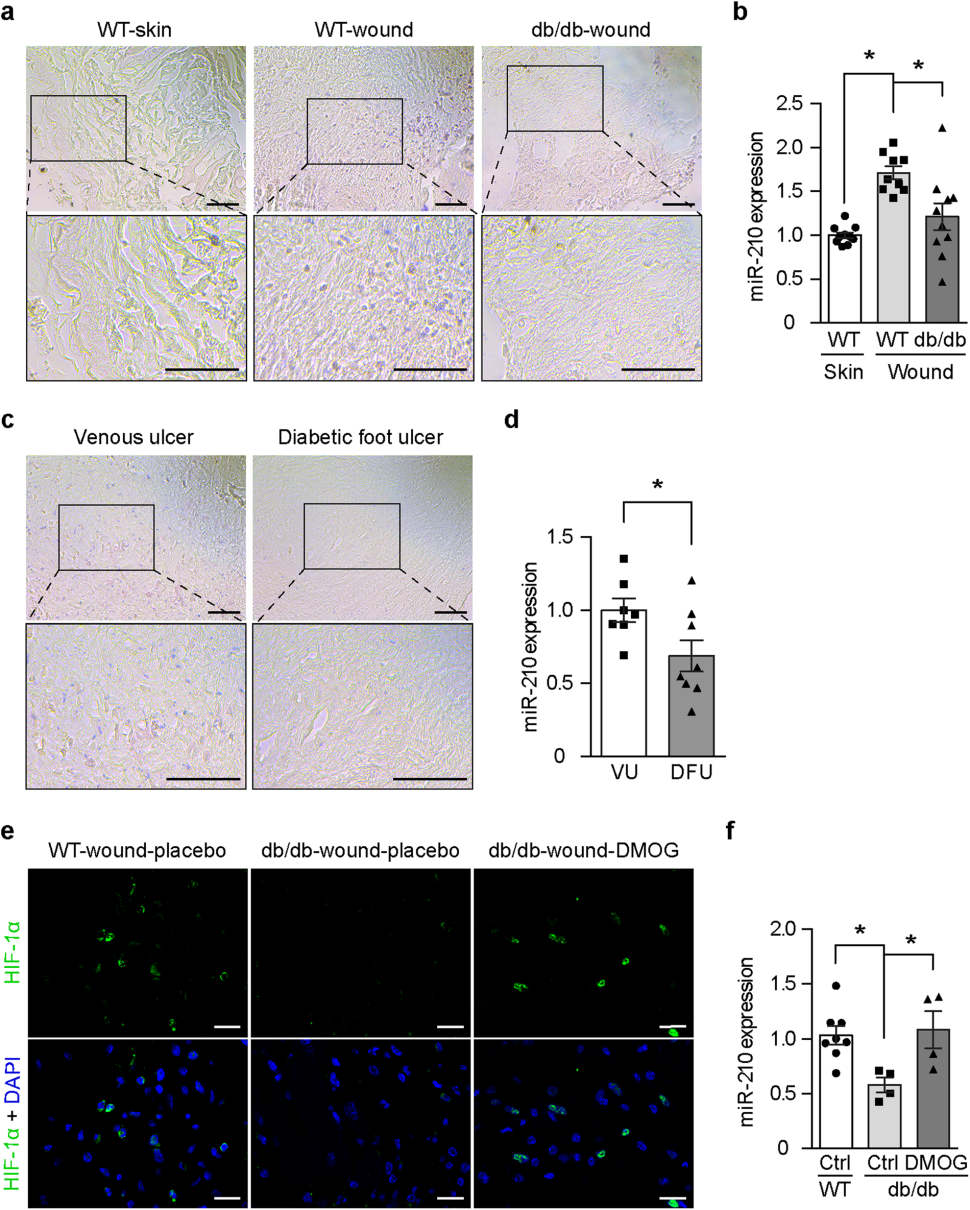
Reduced miR-210 expression in diabetic wounds. **a**, **b** miR-210 expression levels were analyzed in skin (*n* = 10) and wounds (*n* = 9) from normoglycemic wild-type (WT) mice, as well as in wounds from db/db diabetic mice (*n* = 10) using in situ hybridization (**a**) and quantitative RT-PCR (**b**). **c**, **d** Wound biopsies from diabetic foot ulcers (DFU, *n* = 8) and venous ulcers (VU, *n* = 7) were analyzed for miR-210 expression using in situ hybridization (**c**) and quantitative RT-PCR (**d**). Scale bar = 50 μm. HIF-1α expression was analyzed by immunofluorescence (**e**) and miR-210 expression levels by quantitative RT-PCR (**f**) in wounds from WT-control mice (*n* = 8) and wounds from db/db mice treated with placebo (*n* = 4) or with DMOG (*n* = 4). Scale bar = 100 μm. Statistical differences were calculated using one-way ANOVA (**b**, **f**) and Student’s *t* test (**d**). Data are represented as mean ± s.e.m. *P < 0.05.

In response to the hypoxic environment, wound miR-210 expression was increased compared with that of skin in normoglycemic mice, as shown by both in situ hybridization (Fig. 2a) and quantitative RT-PCR analysis (Fig. 2b). The db/db mouse model is a widely validated model for investigating the impaired mechanisms of wound healing in diabetes^11^, in which the hypoxic environment was confirmed^1^ exactly as in humans^3^. Although wounds of db/db diabetic mice are more hypoxic than those of normoglycemic mice^1^, miR-210 expression was significantly lower in the wounds of db/db mice (Fig. 2a, b). These repressed levels of miR-210 reflect the impaired HIF-1 signaling in diabetic wounds^1^ since they could be reversed when HIF-1 signaling was restored by local treatment with Dimethyloxalylglycine (DMOG) known to be able to overcome the repressing effect of diabetes on HIF-1 signaling^2^ (Fig. 2e, f). Moreover, miR-210 expression was reduced in DFU compared to age-matched nondiabetic venous ulcer (VU) subjects (Fig. 2c, d). Taken together, these results suggest that reduced miR-210 expression may contribute to the impaired wound healing in diabetes.

### Local reconstitution of miR-210 in the wounds improves wound healing specifically in diabetes

**Fig. 3:**
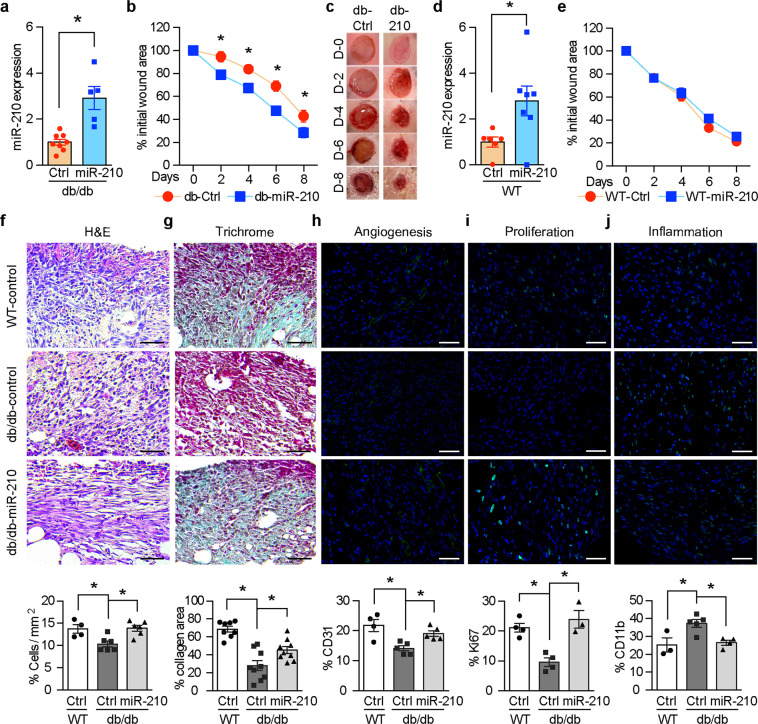
Local miR-210 administration improves wound healing specifically in diabetes. Full-thickness excisional wounds were made on the dorsum of db/db and WT control mice, and control mimic (Ctrl) or miR-210 mimic (miR-210) was injected intradermally in the wound edge on day 0 and day 6. Wounds were harvested on day 8. **a**, **d** miR-210 expression in wounds analyzed by quantitative RT-PCR (*n* = 6, 7, 8, 5 for WT-Ctrl, WT-miR-210, db/db-Ctrl and db/db-miR-210 groups, respectively). **b**, **e** Wound images were obtained every alternate days and wound healing rate is shown as the percentage of the initial wound area (*n* = 10, 11, 12, 14 for WT-Ctrl, WT-miR-210, db/db-Ctrl and db/db-miR-210 groups, respectively). **c** Representative wound images during the healing process. **f**–**j** Levels of granulation, collagen deposition, proliferation, angiogenesis and inflammation were evaluated by histological analysis of H&E staining (**f** *n* = 4, 6 and 6 for WT-Ctrl, db/db-Ctrl and db/db-miR-210 groups, respectively), Masson−Goldner Trichrome staining (**g** *n* = 8, 9 and 9 for WT-Ctrl, db/db-Ctrl and db/db-miR-210 groups, respectively), CD31 (**h** *n* = 4, 5 and 5 for WT-Ctrl, db/db-Ctrl and db/db-miR-210 groups, respectively), Ki67 (**i** *n* = 4, 4 and 3 for WT-Ctrl, db/db-Ctrl and db/db-miR-210 groups, respectively), and CD11b staining (**j** *n* = 3, 5 and 4 for WT-Ctrl, db/db-Ctrl and db/db-miR-210 groups, respectively). Results of semiquantitative evaluations are presented in the histograms. Statistical differences were calculated using Student’s *t* test (**a**, **d**), two-way ANOVA (**b**, **e**) and one-way ANOVA (**f**–**j**), respectively. Data are represented as mean ± s.e.m. *P < 0.05.

In order to examine the pathophysiological relevance of the impaired induction of miR-210 during wound healing in diabetes, we investigated the effects of locally injected miR-210 mimic, a stabilized form of miR-210, on wound healing rate of db/db mice. miR-210 reconstitution promoted wound healing in db/db mice (Fig. 3a–c) despite persistent profound hyperglycemia (Supplementary Fig. 1a, b). The positive effect of miR-210 on wound healing was driven locally since practically no systemic distribution was detected (Supplementary Fig. 1c). Interestingly, miR-210 improved the wound-healing rate specifically in diabetic mice, with no effect on the wound-healing rate in nondiabetic mice (Fig. 3d, e).

The histological examination of the wounds revealed that miR-210 improved several cellular processes essential for wound healing. The overall increase in the granulation tissue (Fig. 3f) followed an increase in the cellular proliferation rate as evaluated by ki67 (Fig. 3i) and angiogenesis as evaluated by CD31 immunostainings (Fig. 3h), which were characteristically impaired during wound healing in diabetes. miR-210 also increased collagen deposition in the granulation tissue of diabetic wounds as assessed by the Masson–Goldner Trichrome staining (Fig. 3g). The prolonged inflammatory phase characteristic of diabetic wounds, denoted by an elevation in the CD11b expression, was also normalized by miR-210 reconstitution (Fig. 3j).

### miR-210 restores metabolic balance in diabetic wounds

**Fig. 4:**
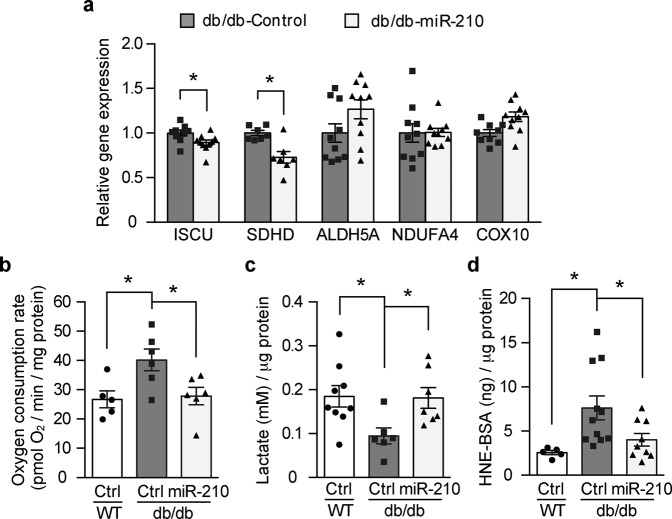
miR-210 restores metabolic balance in diabetic wounds. **a** Gene expression of mitochondrial targets of miR-210—*ISCU, SDHD, ALDH5A*, *NDUFA4* and *COX10* were analyzed from control mimic or miR-210 mimic injected db/db wounds (*n* = 7–10). **b** Oxygen consumption rate was analyzed from freshly harvested wounds using Seahorse XF Analyzer (*n* = 5, 6 and 6 for WT-Ctrl, db/db-Ctrl and db/db-miR-210 groups, respectively). **c** Lactate levels were detected from tissue lysates (*n* = 9, 6 and 7 for WT-Ctrl, db/db-Ctrl and db/db-miR-210 groups, respectively). **d** ROS levels were measured by detecting the amount of 4-HNE in the control mimic or miR-210 mimic injected wounds (*n* = 5, 11 and 9 for WT-Ctrl, db/db-Ctrl and db/db-miR-210 groups, respectively). Statistical differences were calculated by one-way ANOVA. Data are represented as mean ± s.e.m. *P < 0.05.

Taking into account the aforementioned effects of miR-210 on cellular processes that require high energy and the known effect of miR-210 on the energy metabolism^12^, we decided to explore the role of miR-210 on the cellular metabolism during wound healing. Indeed, among the five validated potential mitochondrial target genes of miR-210^13^, local administration of miR-210 repressed the expression of two genes that are central for mitochondrial function, namely *Iron-sulfur cluster assembly enzyme* (*ISCU*) and *D-subunit of Succinate dehydrogenase* (*SDHD*) (Fig. 4a).

In agreement with its effects on these mitochondrial target genes, the administration of miR-210 had a profound influence on mitochondrial respiration by reversing the increased oxygen consumption rate (OCR) in db/db wounds to the levels found in the wounds from control nondiabetic mice (Fig. 4b). Interestingly, miR-210 reconstitution also led to an increase in the production of lactate (Fig. 4c), denoting an increase in glycolysis. Although this metabolic reprograming did not result in a net increase in the ATP production (Supplementary Fig. 2), it contributed to reduced ROS levels in db/db wounds after local miR-210 mimic administration (Fig. 4d).

### Metabolic reprogramming by miR-210 improves fibroblast function

**Fig. 5:**
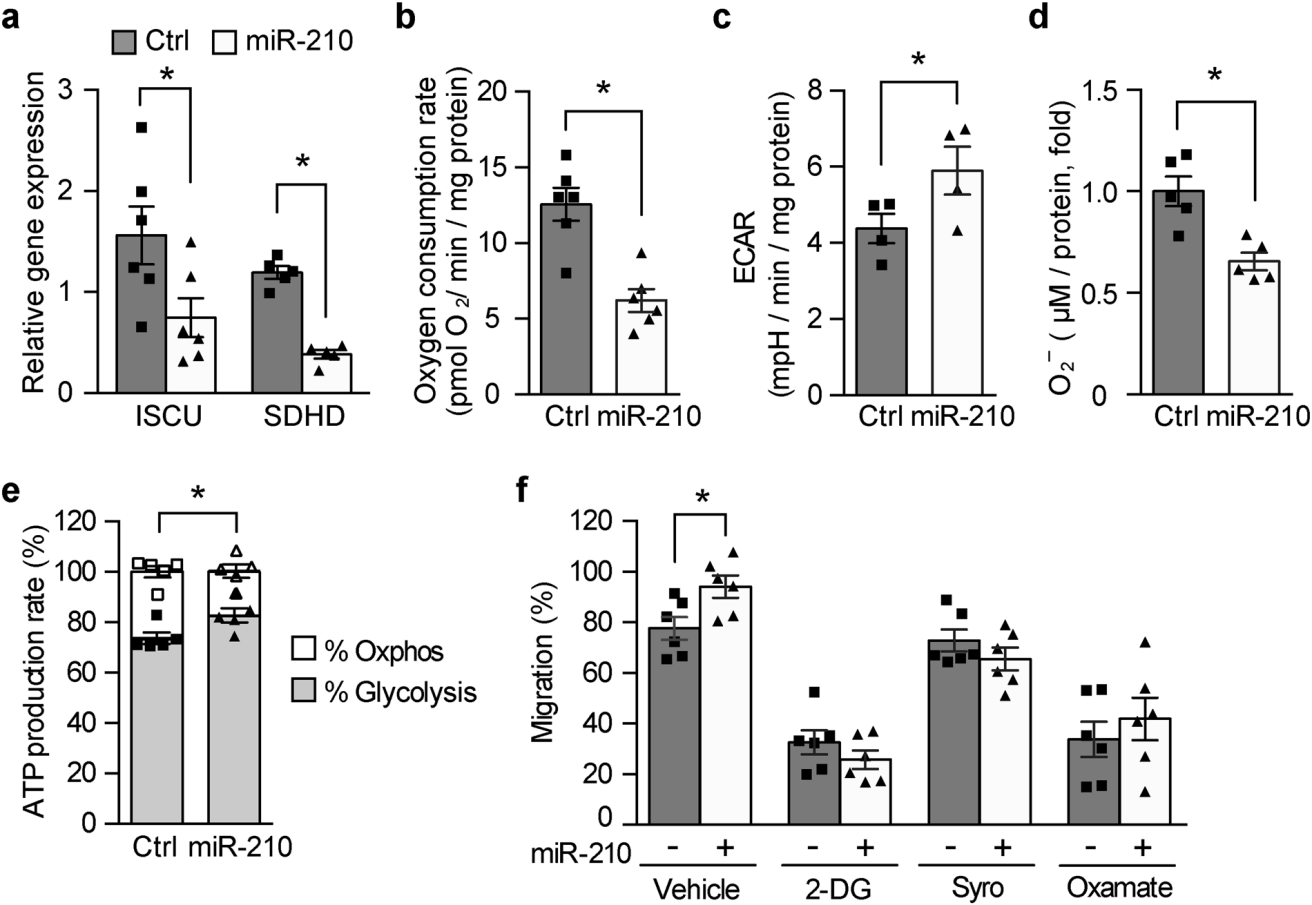
Metabolic reprogramming by miR-210 improves fibroblasts function. HDF cells were transfected with control mimic or miR-210 mimic and exposed to hypoxia and high glucose concentration (30 mM) followed by measurement of **a** gene expression levels of *ISCU* (*n* = 6) and *SDHD* (*n* = 5), **b** oxygen consumption rate (OCR) (*n* = 6), **c** extracellular acidification rate (ECAR) (*n* = 4), **d** ROS levels (*n* = 5) and **e** mitochondrial and glycolytic ATP production rate (*n* = 5). **f** Migration of HDF that were treated with vehicle or glycolysis inhibitors 2-deoxy-d-Glucose (2-DG, 15 mM), Syrosingopine (Syro, 10 µM), and Oxamate (45 mM) along with hypoxia and high glucose concentrations (*n* = 6). Statistical differences were calculated using paired Student’s *t* test. Wilcoxon matched-pairs signed rank test was used for Syrosingopine-treated groups where the data were not normally distributed. Data are represented as mean ± s.e.m. *P < 0.05.

Since the bulk of the granulation tissue consists of fibroblasts, it was of interest to study the effects of miR-210 in these cells in diabetic setting. In accordance with the findings in db/db wounds, miR-210 mimic transfection in HDFs exposed to hypoxia and high glucose concentrations led to a significant decrease in the mRNA levels of *ISCU* and *SDHD* (Fig. 5a and Supplementary Fig. 3a). Moreover, miR-210 reconstitution decreased OCR (Fig. 5b), enhanced glycolysis (Fig. 5c) and diminished ROS levels (Fig. 5d) in HDFs exposed to hypoxia and high glucose levels. The metabolic changes induced by miR-210 reconstitution was further validated by the real-time ATP synthesis rate assay on a Seahorse XF analyzer. As shown in Fig. 5e, a significantly smaller proportion of ATP was generated via mitochondria respiration and a higher proportion of ATP was generated by glycolysis after miR-210 reconstitution in HDFs that were exposed to high glucose levels in hypoxia. We have further investigated the functional implications of the miR-210-induced metabolic reprogramming by evaluating the migration of HDF. The miR-210-induced migration of HDF exposed to high glucose levels in hypoxia was abrogated when glycolysis was inhibited in different ways by, namely, 2-deoxy-d-glucose (2-DG), syrosingopine and sodium oxamate in HDFs (Fig. 5f and Supplementary Fig. 3b). This suggests that the positive effect of miR-210 on HDF migration is mediated by an increase in glycolysis. Taken together, these results indicate that miR-210 could reprogram the cellular energy metabolism which in turn improves the cellular function under diabetic conditions.

## Discussion

The high cellular energy demands during wound healing depends on an appropriate metabolic shift to cope with the low oxygen levels^14^. A pivotal role for the adequate reaction to hypoxia and for wound-healing processes is played by HIF-1 signaling that is responsible for the adaptation of metabolism, cellular motility and proliferation, immune response and angiogenesis^15^. DFU are however characterized by a repressed HIF-1 activation with direct negative consequences for wound healing^3^. Reactivation of HIF-1 signaling in diabetic wounds is sufficient to reverse the wound-healing potential^1^. A clinical study that addresses the effects of activation of the HIF-1 signaling in DFU is ongoing (ClinicalTrials.gov Identifier: NCT03137966). The targets of HIF-1 signaling is however broad and modulate several cellular pathways that might not be involved in wound-healing process. It is therefore of value to explore a narrower relevant hypoxic signature for just the processes important for wound healing. Here we show that miR-210, which directly regulates approximately 30 genes^13^, is sufficient to improve the impaired wound healing specifically in diabetes.

The induction of miR-210 by hypoxia is in perfect agreement with its exclusive regulation by HIF-1 signaling and its ubiquitous expression^6,7^. The central regulatory role of HIF-1 for miR-210 function explains the repressive effect of hyperglycemia on miR-210 induction in hypoxia observed in cells and in vivo, which mirrors the well-documented glucose-dependent suppression of HIF-1 signaling in hypoxia^16^. Low expression of miR-210 has been reported in the pancreatic islets of diabetic animals and contributes to beta cell death^17^. On the other hand, high levels of miR-210 were reported in the serum and urine of patients with newly diagnosed Type 1 Diabetes^18–20^, and the hearts of diabetic rats^21^, but the levels were not corrected for tissue oxygen tensions that are generally lower in diabetic tissues^16,22^. Similar to HIF-1α expression, the miR-210 expression in diabetic tissues is dependent on the balance between the induction by hypoxia and the inhibition by high glucose levels. It is not surprising to detect higher miR-210 levels in diabetic tissues since they are more hypoxic than the corresponding controls which are not hypoxic. The inhibitory effects of high glucose concentrations will be unveiled when comparing hypoxic conditions with similar oxygen tensions or after correction for tissue oxygen concentrations. Our results showed that miR-210 expression increased in response to hypoxia in control wounds but was inhibited by hyperglycemia in diabetic wounds despite a more profound hypoxia^1^. This is confirmed by the fact that miR-210 increases to normal levels when impaired HIF-1 signaling in diabetes is rescued by the local treatment with DMOG. This confirms the insufficient adaptive response to hypoxia in diabetic wounds, which contributes to impaired wound healing.

The characteristically glucose-dependent impairment of cellular functions central for wound healing such as repressed proliferation, migration, and angiogenesis and increased inflammation were at least partially attributable to the decreased miR-210 expression in diabetic wound since its reconstitution restored them. miR-210 reconstitution improved granulation, proliferation and angiogenesis in the same way as the induction of HIF-1 signaling does in diabetic wounds^1^. The positive effect of miR-210 on angiogenesis is in agreement with previous reports showing miR-210 modulates endothelial cell response^23^ and promotes angiogenesis in ischemic tissues^24,25^. The stimulatory effects of miR-210 on cell proliferation and migration are in line with previous observations in normal or transformed cells^23,26–28^. However, this effect seems to be dependent on cellular context and on oxygen levels^9,29^.

The reconstitution of miR-210 in the wounds of diabetic mice was sufficient to improve healing despite persistent hyperglycemia. It is highly specific for diabetic condition probably because of the disease-dependent inhibition of miR-210 during diabetic wound healing but it has no effect when it is expressed at sufficient levels during normal wound healing. Extremely high expression of miR-210 can even have a retarding effect on wound healing since it mimics profound ischemic conditions that are notorious for their deleterious effects on tissue regeneration^9^. It is interesting that inhibition of miR-210 was also followed by improvement in wound-healing rate in a model of impaired healing in diabetes^30^ The apparently opposing effects of miR-210 might be due to the differences in pharmacokinetics and wound models but might also mirror the fact that the biological effects of the hypoxamiR-210 are a mixture between its direct effects and the indirect effects played through HIF-1, which might be differently modulated by different pharmacological approaches.

The beneficial effects of miR-210 on the improvement of wound healing in diabetes can be at least partially explained by its effects on energy metabolism. The OCR of diabetic wounds was highly increased compared with nondiabetic wounds, which is in accordance with other reports on diabetic tissues^31,32^. Increased oxygen consumption results in hypoxia in diabetes in general^22^, and especially in diabetes wounds^1^ due to the mismatch with oxygen delivery. It might well activate a vicious circle since the central mechanism for the adaptive responses to hypoxia, HIF-1 signaling, is repressed in diabetes^16^. Our data demonstrate that miR-210 diminished OCR in diabetic wounds as well as in HDFs exposed to high glucose concentrations in hypoxia. miR-210 could achieve this by downregulating *ISCU*, which is required for the formation of iron-sulfur cluster that is present in various enzymes of the tricarboxylic acid (TCA) cycle. miR-210 also reduced the expression levels of *SDHD*, an enzyme that takes part in both TCA cycle and the electron transport chain (ETC) in the mitochondria. Moreover, miR-210 could enhance glycolysis that was inhibited in diabetic compared with nondiabetic control wounds. ROS levels were diminished by miR-210 as a consequence of the restoration of the balance between mitochondrial respiration and glycolysis. The enhanced glycolysis mediated the positive effects of miR-210 on the migration of HDF that were exposed to high glucose levels in hypoxia. This is in perfect agreement with the emerging role of glycolysis in promoting angiogenesis^33^ and other cellular processes involved in tissue repair and regeneration^34,35^.

**Fig. 6:**
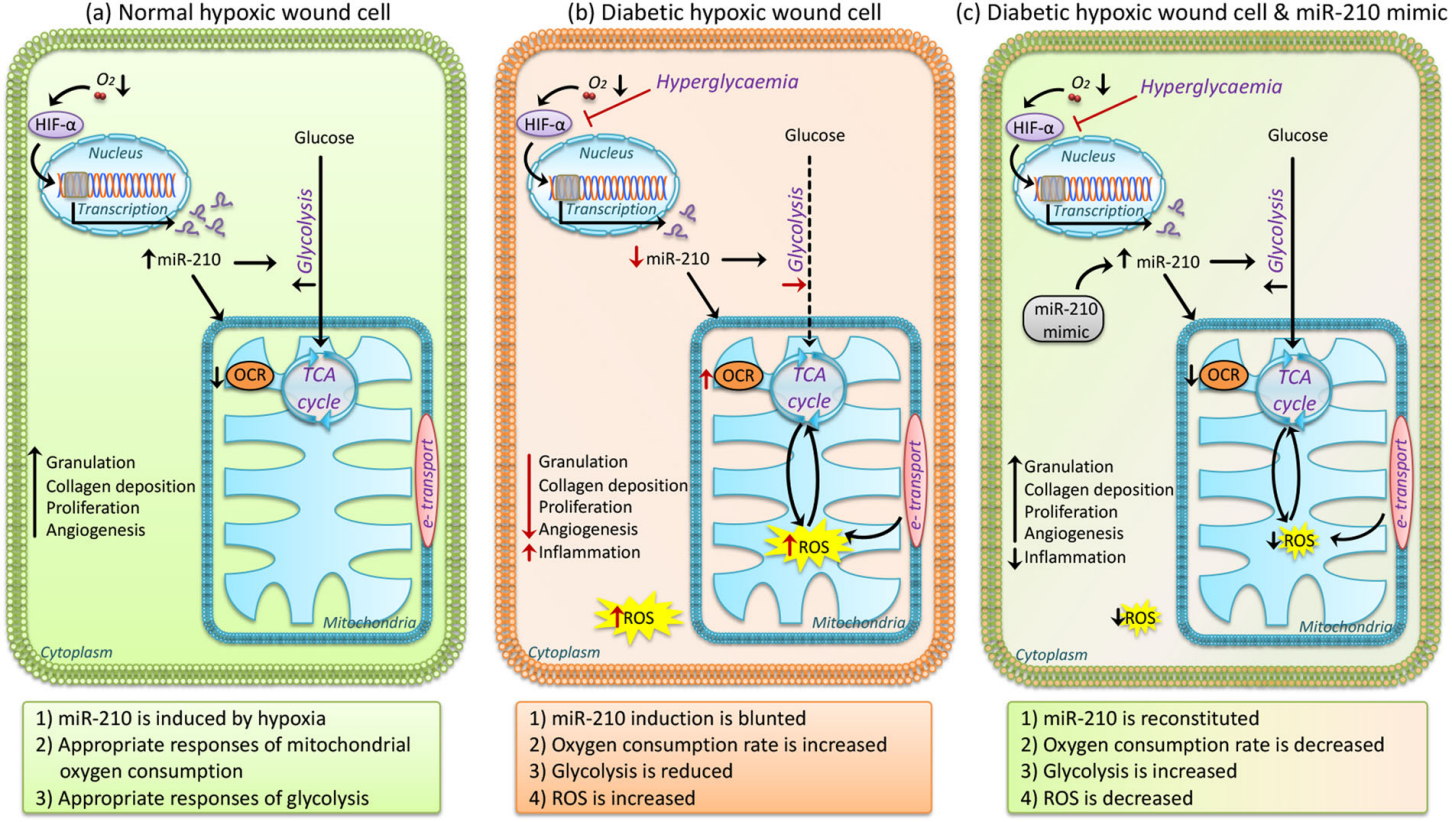
Schematic illustration of the cellular metabolic changes in diabetic wounds that can be reversed by miR-210 reconstitution. **a** In normal hypoxic wound cell, miR-210 is induced by hypoxia and regulates appropriate responses of mitochondrial oxygen consumption (OCR) and glycolysis. **b** In diabetic hypoxic wound cell, miR-210 induction is blunted, resulting in increased OCR, reduced glycolysis, and consequently elevated ROS levels which are detrimental to wound healing. **c** In miR-210-reconstituted diabetic hypoxic wound cells, miR-210 can reverse the metabolic changes by inhibiting OCR and enhancing glycolysis and consequently normalize ROS levels which are beneficial for wound healing.

In conclusion, our results demonstrate a metabolic change in diabetic wounds characterized by increased OCR and ROS levels and decreased glycolysis due to inhibited miR-210 expression by hyperglycemia. miR-210 reconstitution can reverse the metabolic changes by inhibiting OCR and enhancing glycolysis and consequently normalize ROS levels and promote wound healing specifically in diabetes (Fig. 6). These data suggest that local miR-210 administration in diabetic wounds is a promising therapeutic approach for DFU.

## Methods

### Chemicals

Custom-stabilized miRIDIAN mmu-microRNA-210-3p mimic (C-310570-5) and custom-stabilized miRIDIAN negative control mimic #1 (CN-001000-01) were obtained from Dharmacon. MaxSuppressor^TM^ In Vivo RNA-LANCEr II was from BIOO Scientific, RNA*later*^TM^ Stabilization solution were obtained from ThermoFisher Scientific, 10% neutral buffered formalin solution from Sigma, and Mayers Hemotoxylin and 0.2% Eosin from Histolab. Mmu-miR-210-3p-specific LNA-probes were obtained from Exiqon. 2-deoxy-d-Glucose, Syrosingopine and sodium oxamate were purchased from Sigma Aldrich.

### Human wound biopsies

Eight patients with DFU (age: 69.1 ± 9.9 years old; HbA1c: 59.71 ± 11.52 mmol/mol) and seven patients with venous ulcer (age: 78.4 ± 6.9 years old) were recruited after agreeing and providing their informed consent. Detailed information of these subjects is given in Table 1. The Regional Ethical Review Board in Stockholm, Sweden approved the study. The wound biopsies were taken using 4 mm biopsy punch after local anesthesia.

**Table 1:** Information of patients with diabetes and controls.

Donor	Group	Gender	Age	HbA1c (mmol/mol)	Diagnosis	Medication
01	DFU	M	66	45	T2DM, DFU, hypertension, renal failure stage 3, TIA	Metformin, Insulin Aspart, Insulin Glargine, Atenolol, Lisinopril, Fluoxacillin
02	DFU	F	57	63	T2DM, DFU, osteomyelitis, chronic charcot foot	Fluoxacillin, Sitagliptine, Repaglinide, Furosemide, Felodipine, Enalapril, Atorvastatin, Oxycondone
03	DFU	M	60	74	T2DM, DFU, hypertension	Metformin, Insulin Aspart, Insulin Glargine, Atenolol, Lisinopril, Fluoxacillin
04	DFU	M	81	45	T2DM, DFU, dyslipidemia, TIA, CAD, CML	Insulin Aspart, Insulin Protamine, Acetylsalicylic acid, Imatinib, Bisoprolol, Furosemide, Fluoxacillin
05	DFU	M	70	51	T2DM, DFU, renal failure stage 3	Insulin Glargine
06	DFU	M	69		T2DM, DFU, hypertension, dyslipidemia	Insulin Glargine, Metformin
07	DFU	M	86	61	T2DM, DFU, hypertension, dyslipidemia, atrial fibrillation, TIA, CHF, Charcot foot	Insulin Aspart, Insulin Protamine
08	DFU	M	64	79	T2DM, DFU, hypertension, dyslipidemia, renal failure stage 3, COPD	Insulin Glargine, Insulin Aspart, Acetylsalicylic acid, Furosemid, Metoprolol, Enalapril, Simvastatin, Ciprofloxacin
VU1	VU	M	86	—	VU, prostate cancer, depression, cardiac insufficiency, polycythemia vera	Omeprazole, calcium carbonate, and cholecalciferol, Furix, Metoprolol, Losartan, Oxycodone, Paracetamol, Zopiclone, Mirtazapine, Warfarin, Prednisolone, Lactulose, Enanton Depot
VU2	VU	F	78	—	VU, atherosclerosis in extremity arteries	Trombyl, Simvastatin, Oxycodone, Clopidrogel
VU3	VU	F	71	—	VU, atrial fibrillation, venous thrombosis	Calcium carbonate, Potassium chloride, Fragmin, Iron sulfate, Bisoprolol
VU4	VU	F	77	—	VU, skin defect after surgery	Prednisolon, Paracetamol, Tramadol, Calcium carbonate, losartan, Etarnecept
VU5	VU	F	87	—	VU, varices, peripheral atherosclerosis	Natrium chloride, Paracetamol
VU6	VU	M	69	—		Split skin graft
VU7	VU	F	81	—		

*T2DM* type 2 diabetes, *DFU* diabetes foot ulcer, *VU* venous ulcer, *TIA* transcient ischemic attack, *CVD* cardiovascular disease, *COPD* chronic obstructive pulmonary disease.

### Animals

BKS(D)-*Lepr*^*db*^/JOrIRj (db/db) mice and their corresponding normoglycemic wild-type (WT) control mice were obtained from Janvier Labs. The mice were housed up to five per cage in a 12-h light/dark cycle at 22 °C and were provided standard laboratory food and water ad libitum. The animals were caged individually and handled daily for 1 week before wounding. The mice were randomized into groups receiving control microRNA mimic or miR-210 mimic according to their HbA1c levels, body weight and blood glucose levels. The sample size was determined using power calculations based on the results from the same mouse model in our previous studies^1,36^.

### Wound model

The wound model was created as described previously^1^. Following blood glucose control, the mice were anesthetized and the hair from the back were removed using a shaver followed by a depilatory cream. The skin was cleaned with 70% ethanol and two full-thickness wounds extending through the panniculus carnosus were created on either side of the dorsal midline using a 6-mm biopsy punch. MiR-210 mimic (0.125 nmol) or negative control mimic were injected intradermally at four different places in the wound edges on the day of wounding as well as after 6 days post surgery. In wound experiment using DMOG, 100 μL DMOG (2 mM) or vehicle were applied locally every other day. Digital photographs were obtained on the day of wounding and every other day after wounding. A circular reference of known area was placed alongside the wounds to allow for correction of the distance between the camera and the wound. The wound area was calculated using ImageJ software, version 1.47 (NIH, USA), corrected for the area of the reference circle and expressed as percentage of the original area. The data analysis was blinded. On day 8 after wounding, the animals were euthanized and the wounds were harvested. One part of the wounds was snap frozen, one part was placed in RNALater and the rest used for Seahorse analysis. One half of the other wound was placed in 10% formalin to be processed for histology and staining.

### Histology

Histology was performed on formalin-fixed, paraffin-embedded sections (5 µm). The slides were stained with hematoxylin and eosin after deparaffinization and rehydration. Subsequently, the granulation area of the wound was quantified. Image analysis and quantification was done using smart segmentation feature on Image Pro Premier software, version 9.2 (Media Cybernetics). At least two images from each slide were evaluated and each condition had 4–6 slides. Granulation was measured as ratio of the number of cells to the total area of the granulation layer in an image.

### Fluorescent immunohistochemistry

Formalin-fixed, paraffin-embedded tissues (FFPE) were deparaffinized and rehydrated. Antigen retrieval was performed in a microwave (800 W for 10 min) using citrate buffer. Slides were then washed with PBS-T (Phosphate buffered saline supplemented with 0.1% Tween) 3 times for 3 min each. The sections were blocked with goat serum or 5% bovine serum albumin (BSA) in PBS for 30 min at RT, then incubated with primary antibodies overnight at 4 °C. After three times wash with PBS-T for 3 min each, the sections were incubated with fluorochrome-conjugated secondary antibody for 1 h at RT in dark. The sections were washed in PBS-T three times for 3 min each and treated with 0.1% Sudan Black-B solution (Sigma) for 10 min to quench autofluorescence. After washing, the sections were incubated with 1:5000 diluted DAPI (4′,6-diamidino-2-phenylindole, Thermo-Fisher Scientific) in PBS for 5 min and followed by washing in PBS-T for 5 min. The sections were then mounted and stored in 4 °C. The fluorescent images were acquired using Leica TCS SP5 or SP8 confocal microscope (Leica Microsystems).

To detect HIF-1α, the fluorescent signal was enhanced using Tyramide Superboost kit (Thermofisher). Briefly, following antigen retrieval, the sections were blocked with 3% H_2_O_2_ before blocking with goat serum. The sections were incubated with primary antibody and washed with PBS-T 5 min four times followed by incubation with an HRP-conjugated rabbit antibody for 1 h at RT. The sections were washed thoroughly and incubated with tyramide reagent for 10 min at RT in dark. The reaction was stopped by incubating with the stop solution for 5 min and three times washed with PBS-T for 3 min each. The sections were subsequently treated with 0.1% Sudan Black-B solution, followed by counterstaining with DAPI (1:5000) for 5 min. The sections were finally washed with PBS-T and mounted.

Image analysis was performed using Image-Pro Premier, version 9.2 (Media Cybernetics) and ImageJ software, version 1.47 (NIH, USA). For CD31, Ki67 and CD11b staining, 2–5 images per tissue and a total of at least three tissues per condition were considered for image analysis. The total number of nuclei and cells with positive signal were counted. Quantification for each staining is expressed as percentage of positive cells to total number of cells. The following primary antibodies were used: anti-CD31 from Dianova (DIA310, 1:100); anti-Ki67 from Abcam (ab15580, 1:1000), anti-CD11b from Merck Millipore (MABF512, 1:100) and anti-HIF-1α (GTX127309, 1:100). The secondary antibodies used were goat anti-rat Alexa Fluor 488 (A11006, 1:500) and goat anti-rabbit Alexa Fluor 488 (A11008, 1:500) from ThermoFisher Scientific.

### In situ hybridization

For the detection of miR-210 in FFPE sections, Exiqon miRCURY locked nucleic acid (LNA)-DIG labeled probe was used as previously described^37^ with some modifications. Briefly, FFPE slides (5 μm thickness) were deparaffinized and the RNAs were demasked by 15 μg/mL proteinase K treatment for 10 min at 37 °C. The probes were hybridized with Mmu-miR-210-3p-specific LNA-probes (Exiqon) at a concentration of 250 nM for 2 h at 53 °C. Stringent washes were carried out at 53 °C with decreasing concentrations of Saline-Sodium Citrate buffer (SSC) (once in 5× SSC, twice in 1× SSC and twice in 0.2× SSC) for 5 min followed by a final wash with 0.2× SSC at room temperature. The sections were then blocked and incubated with an alkaline phosphatase (AP)-conjugated antibody specific to DIG (Roche, 1:1000) for 1 h at room temperature. The signal for miR-210 was visualized by addition of an AP substrate, NBT/BCIP (ThermoFisher) and the slides were counterstained with nuclear fast red stain (Vectorlabs).

### Masson−Goldner Trichrome staining

FFPE sections of wounds were deparaffinized with two passes in Xylene for 3 min each and rehydrated in sequential passes of 100% and 95% ethanol for 3 min, two times each. The slides were treated according to the manufacturer’s instructions to obtain Masson−Goldner Trichrome staining (Merck Millipore). Images of the staining were obtained using Leica DM3000 LED fluorescence microscope using the transmitted light. The collagen stained areas in the wounds were analyzed and quantified using the Smart Segmentation feature on Image Pro Premier software, version 9.2 (Media Cybernetics). At least 2–4 images from 3 to 4 tissues from each condition were analyzed. Collagen staining was expressed as the percentage of area stained by collagen (green).

### 4-Hydroxynonenal measurement

4-Hydroxynonenal (4-HNE) was measured in frozen wound lysates using commercially available kits (OxiSelect STA-838 and STA-310, Cell Biolabs, San Diego, CA, USA) according to the manufacturer’s instructions.

### Lactate assay

Lactate production was measured using the Lactate Colorimetric Assay kit from Biovision according to the manufacturer’s instructions. Wound tissues were homogenized in lactate assay buffer provided in the kit and lactate levels were measured and normalized to the protein levels.

### Cell culture

Primary HDFs (ATCC, USA) were cultured in Dulbecco’s modified Eagle’s medium (DMEM; 5.5 mM glucose) supplemented with 100 IU/ml penicillin and streptomycin, and 10% heat-inactivated FBS (fetal bovine serum, Invitrogen). The cells were maintained in a humidified atmosphere with 5% CO_2_ at 37 °C in a cell culture incubator, and passages 4–9 were used for experiments. Cells were cultured under normoxic [21% O_2_ (vol/vol)] or hypoxic (1% O_2_) conditions in Hypoxia Workstation INVIVO2 (MedicalExpo).

### Transfection of miR-210 mimic in HDF

HDFs were transfected with miR-210 mimic or negative control mimic using Lipofectamine RNAiMAX (Life Technologies) according to the manufacturer’s instructions. The cells were harvested 24–48 h after transfection. Total RNA was extracted and the expression levels of miR-210 were analyzed.

### RNA purification and quantitative RT-PCR

Total RNA, including microRNAs, was extracted from cells and tissues using a miRNeasy RNA extraction kit (Qiagen). To detect mRNA expression, high-capacity cDNA Reverse Transcription Kit (ThermoFisher Scientific) was used. All quantitative RT-PCR was performed on a 7300 Real-Time PCR System or QuantStudio 6 and 7 Flex Real-Time PCR System (Applied Biosystems) using SYBR Green Master Mix or Taqman Gene Expression Assays (ThermoFisher Scientific). The internal controls for mRNA expression were *PBGD and Actin*. The primer sequences for SYBR Green quantitative RT-PCR were: mouse ALDH5A1_F: CCAGTCATCAAGTTTGATAAGGAG; mouse ALDH5A1_R: GAGCCCTTCATTCACACC; mouse NDUFA4_F: GCATCCCAGCTTGATTCCT; mouse NDUFA4_R: GTTTGCTGTAGTCCACATTCAC; mouse ACTB_F: AAGATCAAGATCATTGCTCCTC; mouse ACTB_R: GGACTCATCGTACTCCTG; mouse PBGD_F: TCCCTGTTCAGCAAGAAGA; mouse PBGD_R: GGCAGTGATTCCAACCAG. TaqMan Gene Expression Assays were Mm02342800_g1 for mouse ISCU; Mm00546511_m1 for mouse SDHD; Mm00617695_m1 for mouse COX10; Mm01143545_m1 for mouse PBGD; and Mm02619580_g1 for mouse β-actin.

### MicroRNA detection

TaqMan microRNA Reverse Transcription kit and TaqMan miRNA assays (ThermoFisher Scientific) were used for the analysis of miR-210, U6 snRNA and snoRNA55 expression (assay IDs were 000512, 001228, and 001973 respectively). U6 was used as internal control for HDF and keratinocytes, and snoRNA55 was used as control for mouse tissues. To detect miR-210 expression in HDMEC and human wounds, cDNA was produced using TaqMan Advanced miRNA cDNA Synthesis Kit (ThermoFisher Scientific) and microRNA expression was detected using TaqMan Advanced miRNA assays (ThermoFisher Scientific), where miR-103a or the average of miR-16, miR-23a, and miR-24 were used as internal controls. The TaqMan Advanced miRNA assays were hsa-miR-210-3p (477970_mir), hsa-miR-103a-3p (478253_mir), hsa-miR-16-5p (477860_mir), hsa-miR-23a-3p (478532_mir), and hsa-miR-24-3p (477992_mir).

### In vitro migration assay

HDF migration was studied using the scratch assay. Cells were plated in 24-well plates coated with collagen (50 µg/µL) overnight and blocked with 3% BSA in PBS for 2 h. At 90% confluency, the cells were transfected with the miR-210 mimic or negative control mimic as mentioned above. At 24 h after transfection, a scratch was generated in each well with a micropipette tip. The cells were rinsed and treated with normal (5.5 mM) or high glucose (30 mM) medium supplemented with 0.2% FBS and the plates were placed in normoxia or hypoxia. Mitomycin C (10 µg/mL) (Roche) was included in the media to prevent cell proliferation. Digital pictures were obtained immediately after scratching and after 16 h using EVOS XL Core Cell Imaging System (ThermoFisher Scientific). Images were analyzed using ImageJ, version 1.47 software (NIH, Bethesda, MD, USA). The experiment was done in triplicates and three images from each replicate were used for analysis. On each image, the distance between the two sides of the scratch was measured at certain intervals using ImageJ and the mean of distance was calculated. Migration rate was calculated as the difference between the mean of distance at 0 h (Distance_0h) and 16 h (Distance_16h) divided by the distance at 0 h ((Distance_0h − Distance_16h)/Distance_0h). The migration rate for each condition was expressed as percentage to the control condition and was expressed as Relative Migration.

### Measurement of oxygen consumption, extracellular acidification, and ATP production rates

Basal OCR, extracellular acidification rate (ECAR), and ATP production rate were measured using XF Cell Mito Stress Test kit, XF Glycolytic Rate Assay kit, and XF Real-Time ATP Rate Assay kit on Seahorse XF Analyzer (Agilent Technologies). The sensor cartridges used for measuring the oxygen flux was equilibrated in an XF Calibrant (Agilent Technologies) for 16–24 h before the experiment in a 0%-CO_2_ 37 °C incubator. The granulation tissue from the wounds taken from mice after 8 days of wounding was carefully dissected and rinsed with unbuffered Krebs-Henseleit buffer (KHB) media. The tissue was placed at the bottom of the XF24 Islet Capture Microplate (Agilent technologies) and covered with a mesh. Four hundred and fifty microliters KHB medium was added to each well containing the tissue and equilibrated in a 0%-CO_2_ incubator for 30 min. The cartridge was then placed on the assay plate and run in the XF analyzer using an optimized protocol to measure basal OCR. For analysis in cells, HDFs were transfected with negative control mimic or miR-210 mimic and treated with normal (5.5 mM) or high (30 mM) glucose levels in normoxia or hypoxia. The cells from each condition were then seeded onto an XF24 or XFe96 Cell Culture Microplate and (Agilent Technologies). The results were normalized to protein concentration or cell number as indicated.

### Electron paramagnetic resonance (EPR) spectroscopy

ROS levels were measured using cyclichydroxylamine (CMH) spin probe and a CP radical standard curve, using EPR spectrometer (Noxygen, Elzach, Germany). HDF cells were transfected with miR-210 mimic/control mimic and were treated with normal or high glucose and hypoxia or normoxia 16 h after transfection. Following 24 h of treatment, media was removed and the cells were washed twice with PBS. Seven hundred microliters CMH buffer (200 μM) was added to the cells and were incubated in a cell incubator for 30 min. The cells were collected in CMH buffer and were frozen in liquid nitrogen prior to measurement.

### Statistics and reproducibility

Statistical analysis and graphing were performed using GraphPad Prism software (version 6). Outliers were identified using Grubbs’ test. Normality of distribution was analyzed using the Kolmogorov–Smirnov test. Differences between two groups were analyzed using two-sided Student’s *t* test for data with normal distribution, and nonparametric test was used for data that was not normally distributed. Multiple comparisons of three or more groups were performed using one-way ANOVA or two-way ANOVA followed by Bonferroni’s post hoc test. *P* < 0.05 was considered statistically significant. All measures were taken from distinct samples, and the sample sizes are presented in figure legends. All the in vitro experiments were performed at least three times independently. Data are presented as mean ± standard error of the mean (s.e.m.).

### Study approval

The experimental animal procedure was approved by the North Stockholm Ethical Committee for the Care and Use of Laboratory Animals. The study using human material was reviewed and approved by the Regional Ethical Committee of Stockholm. Written informed consent was received from participants prior to inclusion in the study. The study was conducted according to the Declaration of Helsinki’s principles.

## Supplementary information

Supplementary information

## Data Availability

The datasets generated during and/or analyzed during the current study are available from the corresponding authors on reasonable request.
