# Supplementary material for: HypoxamiR-210 accelerates wound healing in diabetic mice by improving cellular metabolism
Source: Commun Biol. 2020 Dec 14;3:768. doi: 10.1038/s42003-020-01495-y (PMC7736285; doi:10.1038/s42003-020-01495-y)
Supplement: Supplementary file 1 — Supplementary information [file 42003_2020_1495_MOESM1_ESM.pdf]

Supplementary Information For

HypoxamiR-210 accelerates wound healing in diabetic mice by improving cellular metabolism

Sampath Narayanan[1,2], Sofie Eliasson Angelstig[1,2], Cheng Xu[1,2], Jacob Grünler[1,2], Allan Zhao[1], Wan Zhu[3], Ning Xu Landén[4], Mona Ståhle[4], Jingping Zhang[5], Mircea Ivan[6], Raluca Georgiana Maltesen[7], Ileana Ruxandra Botusan[1,2], Neda Rajamand Ekberg[1,2], Xiaowei Zheng[1,2*†], Sergiu-Bogdan Catrina[1,2,8*†]

[1]Department of Molecular Medicine and Surgery, Karolinska Institutet, Stockholm, Sweden.

[2]Department of Endocrinology and Diabetes, Karolinska University Hospital, Stockholm, Sweden.

[3]Department of Nosocomial Infection Control, China Medical University, Shenyang, China.

[4]Unit of Dermatology and Venereology, Department of Medicine, Karolinska Institutet, Stockholm, Sweden.

[5]Department of Infectious Disease, China Medical University, Shenyang, China.

[6]Departments of Medicine, Microbiology and Immunology, Indiana University, Indianapolis, USA

[7]Department of Anesthesia and Intensive Care Medicine, Aalborg University Hospital, Denmark

[8]Center for Diabetes, Academic Specialist Centrum, Stockholm, Sweden

*To whom correspondence should be addressed:

E-mail: Xiaowei.zheng@ki.se; Sergiu-Bogdan.Catrina@ki.se.

† These authors contributed equally to this work.

This PDF file includes:

Supplementary figure and figure legends: Supplementary Figure 1 – 3.

Supplementary methods

Supplementary figure and figure legends

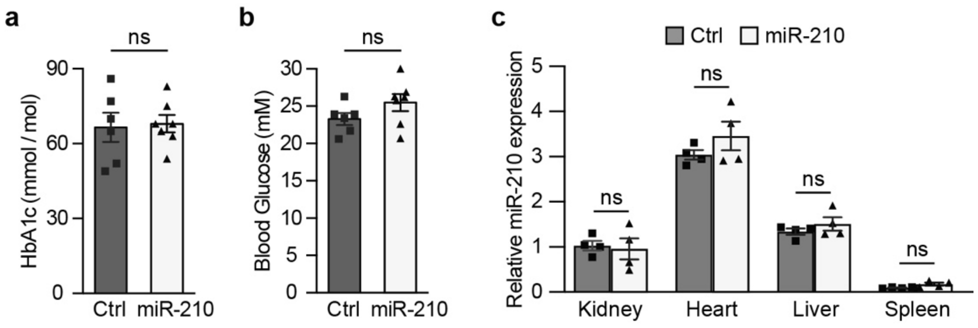

Supplementary Figure 1: HbA1c **(a)** and blood glucose **(b)** were measured from db/db mice for control mimic (Ctrl, n=6) and miR-210 mimic (miR-210, n=7) treatment before wounding. **(c)** Eight days after wounding, kidney, heart, liver and spleen tissues were collected and miR-210 levels were measured (n=4). Statistical differences were calculated using Student's t-test. Data is represented as mean ± s.e.m. No significant difference was found between groups (ns).

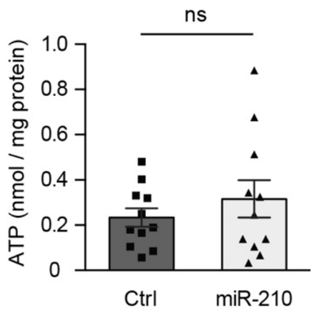

Supplementary figure 2: ATP was measured from wounds of db/db mice injected with control mimic (Ctrl) or miR-210 mimic (miR-210) (n=11) and was normalized to protein concentration**.** Data are represented as mean ± s.e.m. Statistical difference was calculated using Student's t-test. No significant difference was found between groups (ns).

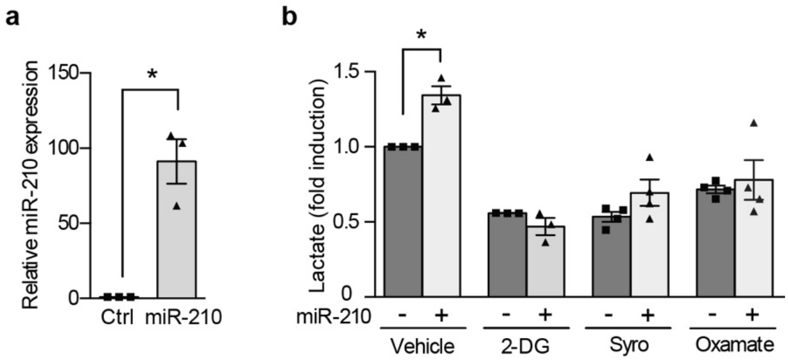

Supplementary figure 3: (a) miR-210 expression was measured by quantitative RT-PCR in HDF cells following the transfection of control mimic (Ctrl) or miR-210 mimic (miR-210) (n=3). **(b)** Lactate was measured in HDF cells transfected with control mimic or miR-210 mimic and was treated with high glucose concentrations (30 mM) in the presence of 2-deoxyglucose (15 mM, n=3), Syrosingopine (10 μM, n=4), Oxamate (45 mM, n=4), or vehicle (n=3) in hypoxia. Statistical differences were calculated by paired Student's t-test. Data is represented as mean ± s.e.m. *, *P<0.05*.

Supplementary methods

Measurement of ATP levels in wound tissue

ATP levels in the wounds were measured using ATP detection assay kit (Cayman Chemical) according to the manufacturer's instructions. Briefly, the tissues were lysed in ATP detection sample buffer and the lysate was added to a reaction mix containing ATP detection assay buffer, D-luciferin and luciferase. Following 20 minutes incubation protected from light, luminescence was recorded in Glomax Luminometer (Promega). The concentration of ATP in the lysates were determined by plotting the absorbance values against serially diluted ATP detection standards. The final ATP levels were finally normalized to protein concentrations.
